# Schooling for children living with human immunodeficiency virus in a community in KwaZulu-Natal, South Africa: Perceptions of educators and healthcare workers

**DOI:** 10.4102/sajp.v76i1.1405

**Published:** 2020-07-22

**Authors:** Stacy Maddocks, Kesni Perumal, Verusia Chetty

**Affiliations:** 1 Discipline of Physiotherapy, School of Health Sciences, University of KwaZulu-Natal, Durban, South Africa

**Keywords:** children, HIV, school, educators, healthcare workers, South Africa

## Abstract

**Background:**

Children living with human immunodeficiency virus (HIV) are faced with challenges, such as social and contextual barriers in society, resulting from their disabilities. Schooling and education, which are crucial for children’s future livelihoods, are areas in which children living with HIV often experience exclusion within South African communities. Educators and healthcare professionals, through collaborative efforts, could influence schooling by improving access and care for children living with HIV.

**Objectives:**

To explore the perceptions of educators and healthcare workers on schooling for children living with HIV in a semi-rural community in South Africa.

**Methods:**

Semi-structured interviews were held, with eight healthcare workers and eight educators, adopting an explorative qualitative approach. Data from the interviews were transcribed and analysed using content analysis.

**Results:**

Four overarching themes were identified: the influence of living with HIV on school readiness and progression; stakeholder support practices to enhance bonding and bridging; obstacles to support; and future directives to foster success at school for children living with HIV.

**Conclusion:**

Educators and healthcare workers felt that social determinants, including poverty and stigma, as well as comorbidities of the virus, influenced the school readiness of children living with HIV. Bonding with children and partnering with caregivers was seen as crucial for fostering successful schooling.

**Clinical implications:**

Additionally, interdisciplinary collaboration between healthcare workers and educators was seen as important for a holistic approach to caring for children living with HIV. Early identification of disabilities was also believed to be important in addressing the social barriers hindering schooling.

## Introduction

‘Human capital is primarily expressed through the combination of two factors: health and education, which are closely linked to each other and to dimensions of inequality’ (Britto 2012:14). Children living with HIV (CLHIV) commonly experience disabilities that span multiple levels including physical impairments such as vision, auditory, musculoskeletal, speech and communication impairments as well as behavioural and learning difficulties that influence children’s social participation (Brassell & Potterton 2019). Children living with HIV experiencing disabilities are not unlike children who experience other non-HIV-related disabilities. However, CLHIV face unique challenges such as living with parents who are ill, which is associated with adverse social and economic consequences as well as the added stigma of being infected with HIV (Steele, Nelson & Cole 2007; Taraphdar et al. 2011). In both instances children with disabilities experience educational and social exclusion which can be addressed by partnerships between education, healthcare and other support and governing structures. These partnerships can assist in achieving optimal learning, social participation and possibly future livelihoods in CLHIV (Rukuni et al. 2018). Children living with HIV are at an educational disadvantage in a number of ways, including school enrolment and attendance; school behaviour and performance; school completion; and educational achievement (Guo, Li & Sherr 2012). Schooling for CLHIV may be interrupted by the effects of the virus, which may lead to physical and cognitive impairment (Brassell & Potterton 2019; Devendra et al. 2013; Rukuni et al. 2018), as well as the social determinants that underpin their realities, such as poverty, stigma, orphanhood and diminished educational motivation caused by absent or detached caregivers (Guo et al. 2012; Toska et al. 2019). Furthermore, the lack of engagement in early childhood development programmes by many CLHIV in resource-constrained settings further undermines their school readiness, resulting in grade repetition and ongoing academic alienation (Zuilkowski et al. 2012).

Schools are recognised as key environments influencing children’s health and well-being (Skovdal & Evans 2017; Toska et al. 2019). The United Nations Educational, Scientific and Cultural Organisation (UNESCO) asserts that schools should provide education, counselling and psychosocial support, and ensure adequate nutrition and healthcare services for children (UNESCO 2008). A large body of research positions schools as having the potential to go beyond the mandate of teaching and learning to address the emotional, physical and social challenges of CLHIV. Whilst the high prevalence of HIV in resource-constrained settings in South Africa puts significant pressure on schools to be sources of support, recent research questions the capability of schools to meet these requirements (Campbell et al. 2016; Skovdal & Campbell 2015).

School educators’ caring abilities are largely influenced by their own socio-emotional status and personal circumstances (Skovdal & Evans 2017). Experts recommend that schools draw on the social capital available to them, such as families, community organisations and government institutions in a multidisciplinary intersectoral collaborative relationship, in order to strengthen the support offered to children living with chronic conditions (Shaw & McCabe 2008). This recommendation corresponds with the idea of an HIV-competent school, as theorised by Campbell et al. (2014). Human immunodeficiency virus competence in schools is premised on offering support to CLHIV in order to address the challenges experienced in the school environment, in collaboration with community stakeholders. Human immunodeficiency virus-competent schools can offer psychosocial pathways through which partners in education provide children with care or social protection. The HIV-competent school seeks to surround the child with support through participation in positive social relationships of school children with their teachers and family (bonding), with community-based institutions of care (bridging) and with non-governmental organisations like funders who are able to lift them out of their social circumstances (linking) (Campbell et al. 2014).

The South African government has developed comprehensive plans to address the needs of children with special needs and disability (which will include CLHIV). These policies include the draft policy on screening, identification, assessment and support (Department of Basic Education 2014) which aims to improve access to education for learners and enhance their participation and inclusion in school. Furthermore the Integrated School Health Policy (National Department of Health and Basic Education 2012) aims to address and improve the general health of children in schools in order to influence their learning. The *Education White Paper 6* on *Special Needs Education: Building an Inclusive Education and Training System* (Department of Education 2001) also provides a guide to improve access and infrastructure for children with special needs (which includes CLHIV) within the South African education system. However, although these policies theoretically offer feasible approaches to address inclusion of CLHIV into basic education within South African systems, there seems to be an apparent gap in translation of policies into daily practice.

In this article, we seek to understand the perceptions of educators and healthcare workers as stakeholders in the care of CLHIV. The article explores the understanding of both professional groups of the readiness of CLHIV for school, as well as their experience of current schooling for CLHIV who may or may not be experiencing disabilities in a community in South Africa.

## Method

Our study used a qualitative design to explore the perceptions of educators and healthcare workers on schooling for CLHIV in a semi-rural community in KwaZulu-Natal (Baxter & Jack 2008). It formed part of a larger umbrella study that aims to assess the feasibility (acceptability, practicality, preliminary efficacy) of an integrated model of rehabilitation and paediatric HIV care in order to improve the diagnosis of, and interventions for, disability in CLHIV. Our study is set at a district healthcare facility and surrounding primary schools which were mainstream, full service schools, and schools for children with special needs. A purposive sample, of healthcare workers involved in the rehabilitative care of CLHIV, as well as educators in the surrounding community schools accommodating CLHIV, was taken (Onwuegbuzie et al. 2009).

Maximum variation sampling was achieved by including male and female participants who had varying experience, professional qualifications and ethnicity.

Semi-structured interviews were conducted at the healthcare facility, and with educators at the neighbouring schools. The interviews lasted between 35 and 45 min. A semi-structured interview guide developed by the authors was used and included open-ended questions about educators’ and healthcare professionals’ perceptions on schooling for CLHIV. Questions included participants’ understanding of HIV and HIV-related disability, and an understanding of South Africa’s policies on disability as well as inclusive education.

Furthermore, questions included participants’ experiences with CLHIV and their school readiness, challenges and facilitators; and also the role of community, healthcare workers and educators in preparing CLHIV for school.

All of the discussions were recorded using a digital voice recorder and conducted in English, as the preferred language of both groups of educators and healthcare workers (isiZulu was also offered as language of choice for interviews). The interviews ceased when no new data emerged from discussions. The interviews were conducted by a member of the research team (one of the authors) with experience in qualitative research approaches. Non-verbal cues were noted by a research assistant who was present if isiZulu was selected as the preferred language of discussion. Data were transcribed immediately following the interviews. Thereafter, an inductive approach of conventional thematic data analysis was conducted (Vaismoradi, Turunen & Bondas 2014). Two authors independently coded the data and agreement, on the overarching themes and subthemes, was reached following in-depth discussions with the third co-author. The methodological rigour was further ensured through member-checking for verification of the results, as well as including rich descriptions of the data in the analysis, and keeping detailed field notes (Creswell & Miller 2000).

## Findings

There were 14 female participants and two male educators who participated, aged between 27 and 61 years. Four participants were black South Africans, five were of Indian descent and six were white South Africans, with one participant of mixed race. The educators were all employed in mainstream primary schools, full service schools, and at schools for children with special needs. Their experience ranged from 4 to 36 years. Two speech therapists, two occupational therapists, two physiotherapists, one psychologist and one dietitian participated. Their experience in healthcare ranged from 1 to 29 years.

Four overarching themes were identified from the interviews with educators and healthcare workers, namely: *the influence of living with HIV on school readiness and progression*; *stakeholder support practices to enhance bonding and bridging*; *obstacles to support;* and *future directives to foster success at school for CLHIV*.

Theme one, *the influence of living with HIV on school readiness and progression*, included the sub-themes of physical and cognitive impairments, social determinants and poor school readiness. Educators and healthcare workers believed that the comorbidities of HIV leading to impairments influenced the school readiness in children living with the virus. Furthermore, social determinants, including poverty, also affected children’s education and schooling.

The second theme, *stakeholder support practices to enhance bonding and bridging*, encompassed the sub-themes of relationship building with CLHIV, partnering with caregivers of CLHIV, stakeholders as problem solvers, and interdisciplinary collaboration. Educators and healthcare workers believed that partnering with CLHIV as well as caregivers was crucial for the successful rehabilitation of schooling practices. Some participants also believed that, as stakeholders, they had to problem-solve around social issues in order to optimise their role in the lives of CLHIV in their care. Additionally, the interdisciplinary collaboration and referral between healthcare workers and educators was seen as important for a holistic approach to caring for CLHIV.

A third theme emerged around *obstacles to support* and included the sub-themes of lack of knowledge about HIV, non-disclosure challenges, delayed disability screening, disparities in mainstream and special needs school environments and gaps in inclusive education policy. Some educators highlighted their lack of understanding of HIV and its influence on children. Both healthcare workers and educators believed that non-disclosure of the virus due to stigma influenced their role in supporting children. Furthermore, the delayed screening of disability in CLHIV was a barrier to schooling and the support offered, as sometimes it resulted in time lost in rehabilitation and education. The disparities between schools for children with special needs and mainstream schools was very apparent to participants and they believed that the policy that guides inclusive education was not translated into practice in the study context.

The final theme, *future directives to foster success at school for CLHIV,* included the sub-themes of early identification of disability, early childhood education and strengthening partnerships through training. Many educators and healthcare workers agreed that early identification of disabilities was important to address the social barriers hindering schooling. Some participants emphasised the importance of early childhood education and the importance of strengthening partnerships through training of all stakeholders, including the community, to foster success for schooling in CLHIV. Table 1 reflects the themes, subthemes and illustrative quotes of educators and healthcare workers.

**TABLE 1:** Themes, subthemes and illustrative quotes of educators and healthcare workers.

Categories	Quotes
**Influence of living with HIV on school readiness and progression**	
Physical and cognitive impairments	‘The child that I’ve taught last year was very thin and very tired all the time. Physically he couldn’t do much of the work. It has affected his work and his working ability, his ability to stay awake in class, his ability to go home and do any work. When I spoke to his father, he said that when he gets home, he sleeps; he doesn’t have the energy to do the work. So, his stamina is impaired, even in physical education lessons he couldn’t manage’. (Educator, main stream school, 6 years experience)
	‘I tend to find that the more wasted (malnourished) a child is, the weaker the child is, the more difficult it is for them to perform daily tasks. A weaker child is a poor-performing child’. (Dietician, health care facility, 7 years experience)
Social determinants	‘You will often see a delay in physical, emotional and social development in these children, which definitely influences their school readiness’. (Psychologist, full service school, 3 years experience)
	‘The parents, just not being able to be there for their children, not reading with them. I mean, I have that problem now. The children can’t read properly because they never hear people reading to them. Nothing makes sense to them. Early stimulation is key’. (Educator, full service school, 29 years experience)
	‘I must say that the impact of the resource deficits comes through – in terms of the availability of somebody to do the appropriate homework, the appropriate carry over, or the lack of materials within the home. It’s very much to do with reading stories, playing games, those kinds of resources, which develop language and develop learning, develop problem-solving, allow for inferencing skills’. (Speech therapist, full service school, 28 years experience)
Poor school readiness	‘Children sometimes have to travel a long distance to go to school and if a child is weak then the child won’t be able to cope with this distance. There will be a hindrance, especially if the parents are poor and they cannot provide transport for the child. This will also increase absenteeism’. (Educator, main stream school, 6 years experience)
	‘Most of our children fail. The academic performance is very weak, but that’s not just the children – there are so many factors that contribute to them failing. They don’t have parents that are going to sit with them and make sure that they do their work: they’re either unemployed, prostituting themselves, or they’re in a tavern somewhere. There is no continuation and parents blame us for not disciplining their child, but discipline starts at home. It’s just difficult’. (Educator, main stream school, 6 years experience)
	‘We work with children who haven’t been able to hear their whole life and this has caused them to be so delayed in their school progress because they haven’t been hearing anything. They still don’t know how to read or write’. (Speech therapist, health care facility, 1 year experience)
**Stakeholder support practices to enhance bonding and bridging**	
Relationship building with CLHIV	‘The big thing is knowing your children, that’s why every day I try and make some sort of connection with them, regardless of what it’s about – just start a conversation with them so that you know what their attitude towards the day is. For example, a student’s parents had to go away; he was left alone with his sister. If I hadn’t started that little conversation with him, I wouldn’t have known. This whole week he has been so upset, crying at the drop of the hat, hasn’t done his homework. If you don’t actually know what’s going on in their little lives, there’s no way that you can give them the coping mechanisms or the extra support that they are going to need. I think having a personal relationship allows you to rectify things before they become a big issue’. (Educator, school for children with special needs, 28 years experience)
Partnering with caregivers of CLHIV	‘I try to get to know the children, especially the children with illnesses like HIV or children with disabilities, and see how I can help because teaching isn’t just being able to get them to understand a concept or subject but getting [to] them engage in their learning. It’s not easy, but I feel like if I don’t develop a good relationship with the kids, I won’t get any knowledge into them’. (Educator, full service school, 4 years experience)
Stakeholders as problem solvers	‘Engaging with the parents at teacher-parent meetings leads to a little insight into their everyday lives, which has changed how I deal with some of the children. In the past I would get irritated when they didn’t do their homework, but then when I have a bit more insight I realise that dad works all day and part-time at night, so the poor kid is there all day alone at home looking after his little sister. So then you go a little bit easier on him and you help him a little more before he leaves school so that he can understand it better when he gets home. For me, I have them all on WhatsApp. I’ve made a group for all the parents, having them all together in a group makes communication so much easier’. (Educator, school for children with special needs, 28 years experience)
Interdisciplinary collaboration	‘I try to get to know the caregiver and also get involved in the other aspects of the child’s life, not just prescribe exercise or give them a home programme. I try to get the mother or father to be part of the total rehabilitation of the child I work with’. (Physiotherapist, health care facility, 12 years experience)
	‘If they don’t have food or things like that, I will provide, but I noticed that they have no appetite. If there is a homework issue, we make time at school. Even if it means staying back during breaks, I will do it if need be’. (Educator, school for children with special needs, 28 years experience)
	‘I often see other issues with children with HIV in my school. They are weak because they not eating, or other kids are bullying them. So I try and help them to get a better diet or address the bullying issue. It’s my duty as an educator as they are in my care’. (Educator, full service school)
	‘He was not coping at school, memory wise. He felt discouraged to a point where the aunt wanted to remove the child from that school, and I managed to help. He was limping when he walked, so I had to treat the child as well as educate the aunt. According to my assessment, he presented with peripheral neuropathy, so I had to assist with that and treat that condition. I also had to help with locating him in another school by collaborating with the principal of the alternate school until he was accepted there’. (Physiotherapist, health care facility, 12 years experience)
	‘The occupational therapist, the speech therapist, the physiotherapist, everyone together working towards the well-being of the child. I always thought that the multi-disciplinary team was just medical, but I have come to realise that I am also a part of the team. It’s the child in the middle and everyone around them’. (Educator, school for children with special needs, 28 years experience)
**Obstacles to support**	
Lack of HIV knowledge	‘I have no idea about the extent of HIV in children. We did, obviously, touch on it in varsity – just the parent being too sick to work, and then the children being negatively affected, not being able to go to school, not having enough food when they went to school etc.’. (Educator, school for children with special needs, 4 years experience)
	‘I’m not too aware of the HIV policies here at school but I know that they are there if I need them, and our HOD is brilliant, so she assists with anything that may arise’. (Educator, main stream school, 6 years experience)
Non-disclosure challenges	‘Disclosure is a huge challenge that needs to be overcome. In many cases, we had to speak to the carers and the foster parents because, for them, it was their stigma about the condition that stopped them from telling the child. There was also their fear of the child being stigmatised should other children find out. Stigma still exists and that’s a challenge that children often face’. (Psychologist, full service school, 3 years experience)
	‘I only found out his status towards the end of the year, so I had no idea what was going on until his father finally disclosed his status… If we know the status earlier, we can be more alert to these disabilities and assist the child more’. (Educator, main stream school, 6 years experience)
Delayed disability screening	‘There is a lack of screening of these children with regards to disabilities. When they go to the clinics or to the doctors, some of their problems are not picked up because they are not screened such that it (referring to disabilities) is only discovered when it is in a late stage’. (Physiotherapist, health care facility, 12 years experience)
	‘We often find that a lot of children have come in recently for screenings to see if we are a suitable placement for the child and they are coming in as six- or seven-year olds having had very little interventions since they left the hospital and there is this big gap in development. Where have the children been?’ (Educator, school for children with special needs, 4 years experience)
Disparities in mainstream and special needs school environments	‘Everything is so flat due to it being wheelchair friendly, so it makes it so much easier for the children to get around. We have the rails, so everyone feels safer, more protected. Everything is absolutely accessible. Children go to computers, they go to a reading room, so over-and-above what we do in a classroom, they have so much external activities that aid in their stimulation. Children have the choice to do sports like the nature reserve, or cross country, and they are encouraged to interact with each other safely during breaks as well. We have reading classes, chess, table tennis. There is a good variety’. (Educator, school for children with special needs, 28 years experience)
	‘We don’t have any luxuries – swimming pool, tennis courts, library. We barely have the basics. The child needs to be able to get around the school independently. A child in a wheelchair can’t come to our school because there will be no way to get around’. (Educator, main stream school, 6 years experience)
Inclusive education policy gaps	‘The whole idea of the *White Paper 6* is quite idealistic, while we find that quite a few children cannot even cope with our academic system and there’s nowhere really for them to go’. (Educator, main stream school, 6 years experience)
	‘Every school has to be prepared and resourced so that it allows for people with disabilities to become a part of that school and that’s where most schools are lacking. If you go to a mainstream school now, you’re not going to find handlebars and rails, or markings on the floor; nor will you find specialised toilets for the disabled child. The *White Paper* is well and good, but then our schools need to be better equipped to carry out this mandate’. (Educator, full service school, 29 years experience)
**Future directives to foster success at school for CLHIV**	
Early identification of disability	‘I think advocating for those early assessments and creating policies in hospitals where we say all our children with HIV need to come be developmentally screened by the entire rehabilitation team is important’. (Physiotherapist, full service school, 6 years experience)
	‘The hospital or clinics need to identify children with HIV needing special care, or those with disabilities, and follow-up on schooling too. But first they need to see if those kids have disability. We don’t see them like physiotherapists and occupational therapists see them. This will help us when we intake. We can be aware of the needs of these children’. (Educator, main stream school, 6 years experience)
Early childhood education	‘Early intervention when the brain is plastic enough, is 0–5. So these children need the appropriate preschool placement and I just feel that nobody acknowledges the role of the preschool and the Grade R year sufficiently enough to get the Grade 1 and 2 going’. (Psychologist, full service school, 3 years experience)
	‘I think we need to identify children with HIV early and make sure they are in school and receiving education and not hidden away. It’s damaging their early years for development’. (Educator, school for children with special needs, 28 years experience)
Strengthening partnerships through training	‘I feel like we need to come together, whomever it may be, and have a more open line of communication for holistic treatment of the child. I think workshops and training would be beneficial if someone would offer that to us! It would make us more aware and help these children, instead of waiting for someone else to do that. We are constantly asking for workshops because they’re so important, but nobody gives them to us. If you guys reach out to us it would be amazing, especially schools like ours. We look for help and we are grateful when that help is given to us, no matter what it may be’. (Educator, main stream school, 6 years experience)
	‘So again, because we are a resourced school we are very happy to provide any kind of training that we can. When we run courses in the afternoon, we invite all the mainstream schools to come and attend. We often take calls from staff at mainstream schools who need help when they’re stuck with an issue; We are very open to helping. It is part of our role within the education system’. (Educator, full service school, 29 years experience)
	‘I think things like community outreach and training would be beneficial. This entire early childhood development programme would be great because they look at the child as a whole – when they are with caregivers, when they are at school, what they are eating. It shouldn’t be just Department of Health, or Department of Education, we should all be collaborating and giving each other the support. We need to work on the social development side of things as well. We need the help of the educators because what they say is gospel; they would be a powerful resource to use’. (Dietician, health care facility, 7 years experience)

### Ethical consideration

The study received full ethical clearance from the University of KwaZulu-Natal Biomedical Research Ethics Committee (Ethical clearance no. BFC 386/17). Prior to commencement of the study gatekeeper approval was sought, as well as informed consent from individual participants. Confidentiality was maintained and participants were informed of their voluntary participation. No incentives were offered throughout the study (Gill et al. 2008).

## Discussion

The study sought to explore the perceptions of educators and healthcare workers on schooling for CLHIV.

The influence of living with HIV on school readiness and progression emerged as a dominant theme. The phenomenon was described by educators and healthcare workers as two-fold: the noticeable functional difficulties experienced by CLHIV, coupled with the social determinants that influenced their school readiness, level of engagement and their progression. Functional difficulties were explained as impairments, both physical and cognitive and participants made reference to visual, hearing and mobility impairments in the children as well as observed weakness, which they viewed as contributing to fatigue and concentration difficulties at school. These findings are corroborated in a recent study on the prevalence of disability in CLHIV in South Africa (Brassell & Potterton 2019). Healthcare workers in our article attributed the cause of these impairments to medical conditions like recurrent otitis media, peripheral neuropathy and malnutrition, whilst the educators placed a larger emphasis on the social determinants such as disengaged caregivers, stigma and the lack of early childhood education leading to poor school readiness and progression in CLHIV. Participants believed that these impairments and contextual barriers challenged the children’s level of participation and performance at school, leading to absenteeism, exclusion in sporting activities and grade repetition.

A speech therapist described a teenager presenting with illiteracy as a result of a missed early childhood hearing impairment. This undetected disability is indicative of the influence that such impairments can have on a child’s learning. The high prevalence of hearing impairments related to HIV (Brassell & Potterton 2019; Devendra et al. 2013; Rukuni et al. 2018) necessitates greater disability screening and awareness in CLHIV, so that appropriate rehabilitation can commence timeously to address these impairments and the associated social barriers placed on children by communities, including schools.

Social constructs, such as poverty and caregiver absenteeism, were factors that resonated with educators and healthcare workers. Such social determinants prompted empathic, responsive adaptions to child learning expectations, requiring educator flexibility in classroom practices. Empathy has been considered the distinguishing feature motivating caring practice amongst educators (Zembylas 2008). Many sub-Saharan African studies have similarly highlighted pastoral acts of care by teachers towards children in need at school (Ogina 2010; Ogina & Ramare 2019); and whilst these caring behaviours are well intended and appreciated, they are often too individual and personalised to promote any long-term care for CLHIV that is sustainable (Campbell et al. 2016; Skovdal & Evans 2017). Research points to the need for a more deliberate, systematic, institutionalised ethic of care to be practised at schools to encourage a sustained supportive school environment for CLHIV (Campbell et al. 2016). Furthermore, a caring school should draw on the social capital resources of all stakeholders amongst whom a school is situated (Khanare 2012).

Despite efforts at maintaining opportunities for open communication and partnerships with caregivers in the community, particular concern was raised by educators at the lack of child HIV status disclosure by parents. Educators observed non-disclosure as a limitation to the degree of care and support they were able to provide to the children. Non-disclosure obstructed occasions for additional support and classroom flexibility and possibly early intervention strategies being successfully implemented. Brainin et al. (2019) found that HIV-related stigma significantly hinders disclosure and in turn limits teachers’ ability to provide care and support. Whilst caregivers understandably fear the possibility of their child being marginalised, they should be enlightened that non-disclosure potentially shuts doors of opportunity for health coverage and social protection referral. Educators need to be aware that caregivers have a right to confidentiality and should be trained to identify the symptoms of children with impairments leading to disabilities, and refer them appropriately to the healthcare cadre.

Despite their expressed desire for child HIV disclosure, the educators admitted to being unfamiliar with the relevant HIV policy adopted at the respective schools, and alluded to particular senior staff members being more knowledgeable. The educators further articulated a lack of HIV-specific education and training and expressed interest in HIV education programmes in the future. Whilst HIV training may bridge a knowledge gap for the educators, African studies have shown that teacher training in HIV education and policy is not synonymous with competent HIV care (Campbell et al. 2015).

Although educator responses from the full service school suggested inclusiveness regarding infrastructure and site-based support teams, as recommended by the *Education White Paper 6* (Department of Education 2001), if our study data were to be analysed against the backdrop of HIV competence (Campbell et al. 2014), both schools may still be lacking in addressing HIV. Schools are considered environments in which social networks occur naturally, making them ideally positioned to offer support to CLHIV (Campbell et al. 2014); but sub-Saharan studies have shown that schools are often poorly equipped to deal with this important challenge, particularly in areas of high HIV prevalence (Campbell et al. 2016; Skovdal & Campbell 2015), such as Kwazulu-Natal, South Africa.

Hoadley (2007) cautioned not to overburden South African educators in resource-constrained schools with additional roles such as supporting and monitoring vulnerable children, when they are struggling to maintain their professional identities in the midst of the teaching and learning crises. Instead she urged that schools be regarded as places from which referral to experts, external to the educators, can be facilitated. This admonition is supported by Skovdal and Campbell (2015) and explains why the mainstream educators’ responses were noticeably charged with despondence and desperate appeals for assistance. Campbell et al. (2016) acknowledge the tremendous importance of caring for the carer (in this case, the educators) by providing them with greater support and strong networks for referral, as well as acknowledgement for the role they play in addressing the needs of CLHIV.

Educators rightly recognised that they are part of the multidisciplinary team. Healthcare professionals expressed a willingness to offer support to educators of CLHIV.

The opportunities for bridging relationships between the educators and the healthcare professionals, as described by Woolcock (2001), were identified by all of the participants, as they believed it was an opportune time for inter-sectoral collaboration and community outreach in addressing the needs of CLHIV. Both groups of participants earnestly stressed the necessity for ongoing engagement in order to strengthen and improve early childhood development, share skills and identify and refer disabilities. Kalembo et al. (2018) affirmed the great benefits accruing from the collaborative training of healthcare workers and educators working with families living with HIV. The shared commitment between both professional sectors would add great value and continuity of care for CLHIV, fostering success in school and within communities.

## Limitations

The perceptions of the educators and healthcare workers in the study are context-specific to a community in KwaZulu-Natal, South Africa, and cannot be generalised to other well-resourced communities in the country.

Furthermore, the perceptions of caregivers, as well as CLHIV themselves, would have added value to our study; but this was not included in the aim of our article.

## Conclusion

The needs of CLHIV are compromised by the social and contextual barriers in the communities where they live. Schooling and school environments are often challenged with issues of access, thus excluding CLHIV from optimal education and reciprocal learning. Amongst the challenges that emerged in our article were a lack of knowledge of HIV and the delayed screening of disabilities in CLHIV, as well as poverty, which influence school readiness and learning. Furthermore, whilst policy in South Africa does address issues of accessibility and inclusion for children with disabilities, a gap exists in translating this into practice. Early identification of disabilities is an important step to address the social impediments limiting schooling for CLHIV. Furthermore early childhood education is also crucial to address the learning needs of CLHIV. Strengthening partnerships through training of all stakeholders, including the community, on CLHIV and their special needs is crucial in the holistic approach to rehabilitative care.

## Recommendations

Further studies need to explore the perceptions of caregivers of CLHIV about schooling. It is also important to consider the perspectives of CLHIV about their own learning. Additional studies will add to the current gap in research and can contribute to integrated models of care offered to children. Additionally, a comprehensive enquiry into the education system in South Africa is important to address the needs of CLHIV and the suitability of the current system.

## References

[CIT0001] Baxter P. & Jack S, 2008, ‘Qualitative case study methodology: Study design and implementation for novice researchers’, *The Qualitative Report* 13(4), 544–559.

[CIT0002] Brainin F., Merrild K., Rueskov V. & Skovdal M, 2019, ‘You need to know in order to help’: How HIV-related stigma obstructs pastoral care in Kenyan primary schools’, *Vulnerable Children and Youth Studies* 19, 1–8. 10.1080/17450128.2019.1668582

[CIT0003] Brassell S.E. & Potterton J, 2019, ‘Prevalence of disability in HIV-infected children attending an urban paediatric HIV clinic in Johannesburg, South Africa’, *Vulnerable Children and Youth Studies* 14(2), 95–115. 10.1080/17450128.2019.1566682

[CIT0004] Britto P.R, 2012, *Key to equality: Early childhood development*, viewed n.d., from http://aserpakistan.org/document/learning_resources/2014/Early_Childhood_Education/Consultative%20Group%20on%20ECCD%20Findings.pdf

[CIT0005] Campbell C., Andersen L., Mutsikiwa A., Madanhire C., Nyamukapa C. & Gregson S, 2016, ‘Can schools support HIV/AIDS-affected children? Exploring the “ethic of care” amongst rural Zimbabwean teachers’, *PLoS One* 11(1), e0146322 10.1371/journal.pone.0146322 26790103 PMC4720431

[CIT0006] Campbell C., Andersen L., Mutsikiwa A., Pufall E., Skovdal M., Madanhire C. et al., 2015, ‘Factors shaping the HIV-competence of two primary schools in rural Zimbabwe’, *International Journal of Educational Development* 41, 226–236. 10.1016/j.ijedudev.2014.05.007 26997748 PMC4793550

[CIT0007] Campbell C., Coultas C., Andersen L., Broaddus E., Skovdal M., Nyamukapa C. et al., 2014, *Conceptualising schools as a source of social capital for HIV affected children in southern Africa*, HCD Working Paper Series, 6, The London School of Economics and Political Science, London.

[CIT0008] Creswell J.W. & Miller D.L, 2000, ‘Determining validity in qualitative inquiry’, *Theory into Practice* 39(3), 124–130. 10.1207/s15430421tip3903_2

[CIT0009] Department of Basic Education, 2014, *Draft policy on screening, identification, assessment and support*, Government Printer, Pretoria.

[CIT0010] Devendra A., Makawa A., Kazembe P.N., Calles N.R. & Kuper H, 2013, ‘HIV and childhood disability: A case-controlled study at a paediatric antiretroviral therapy centre in Lilongwe, Malawi’, *PLoS One* 8(12), e84024 10.1371/journal.pone.0084024 24391869 PMC3877142

[CIT0011] Gill P., Stewart K., Treasure E. & Chadwick B, 2008, ‘Methods of data collection in qualitative research: Interviews and focus groups’, *British Dental Journal* 204(6), 291 10.1038/bdj.2008.192 18356873

[CIT0012] Guo Y., Li X. & Sherr L, 2012, ‘The impact of HIV/AIDS on children’s educational outcome: A critical review of global literature’, *AIDS Care* 24(8), 993–1012. 10.1080/09540121.2012.668170 22519300 PMC8183108

[CIT0013] Hoadley U, 2007, ‘Boundaries of care: The role of the school in supporting vulnerable children in the context of HIV and AIDS’, *African Journal of AIDS Research* 6(3), 251–259. 10.2989/16085900709490421 25866171

[CIT0014] Kalembo F.W., Kendall G.E., Ali M., Chimwaza A.F. & Tallon M.M, 2018, ‘Primary caregivers, healthcare workers, teachers and community leaders’ perceptions and experiences of their involvement, practice and challenges of disclosure of HIV status to children living with HIV in Malawi: A qualitative study’, *BMC Public Health* 18(1), 884 10.1186/s12889-018-5820-z 30012133 PMC6048770

[CIT0015] Khanare F, 2012, ‘Schoolchildren affected by HIV in rural South Africa: Schools as environments that enable or limit coping’, *African Journal of AIDS Research* 11(3), 251–259. 10.2989/16085906.2012.734985 25860099

[CIT0016] National Department of Health and Basic Education, 2012, *Integrated School Health Policy*, NDHBE, Pretoria.

[CIT0017] Ogina T.A. & Ramare N.M, 2019, ‘Accountability of school stakeholders in ensuring orphaned children’s school attendance’, *South African Journal of Childhood Education* 9(1), 1–11. 10.4102/sajce.v9i1.672

[CIT0018] Ogina T.A, 2010, ‘Teachers’ pastoral role in response to the needs of orphaned learners’, *International Journal of Education policy and Leadership* 5(12), 1–10. 10.22230/ijepl.2010v5n12a232

[CIT0019] Onwuegbuzie A.J., Dickinson W.B., Leech N.L. & Zoran A.G, 2009, ‘A qualitative framework for collecting and analyzing data in focus group research’, *International Journal of Qualitative Methods* 8(3), 1–21.

[CIT0020] Rukuni R., McHugh G., Majonga E., Kranzer K., Mujuru H., Munyati S. et al., 2018, ‘Disability, social functioning and school inclusion amongst older children and adolescents living with HIV in Zimbabwe’, *Tropical Medicine & International Health* 23(2), 149–155. 10.1111/tmi.13012 29160948 PMC5814868

[CIT0021] Shaw S.R. & McCabe P.C, 2008, ‘Hospital-to-school transition for children with chronic illness: Meeting the new challenges of an evolving health care system’, *Psychology in the Schools* 45(1), 74–87. 10.1002/pits.20280

[CIT0022] Skovdal M. & Campbell C, 2015, ‘Beyond education: What role can schools play in the support and protection of children in extreme settings?’, *International Journal of Educational Development* 41, 175–183. 10.1016/j.ijedudev.2015.02.005

[CIT0023] Skovdal M. & Evans R, 2017, ‘The emergence of an ethic of care in rural Kenyan schools? Perspectives of teachers and orphaned and vulnerable pupils’, *Children’s Geographies* 15(2), 160–176. 10.1080/14733285.2016.1214236

[CIT0024] South Africa, Department of Education, 2001, *Education white paper 6: Special needs education: Building an inclusive education and training system*, Department of Education, Pretoria.

[CIT0025] Steele R.G., Nelson T.D. & Cole B.P, 2007, ‘Psychosocial functioning of children with AIDS and HIV infection: Review of the literature from a socioecological framework’, *Journal of Developmental & Behavioral Pediatrics* 28(1), 58–69. 17353739 10.1097/DBP.0b013e31803084c6

[CIT0026] Taraphdar P., Guha R.T., Haldar D., Chatterjee A., Dasgupta A., Saha B. et al., 2011, ‘Socioeconomic consequences of HIV/AIDS in the family system’, *Nigerian Medical Journal* 52(4), 250 10.4103/0300-1652.93798 22529508 PMC3329095

[CIT0027] Toska E., Cluver L., Orkin M., Bains A., Sherr L., Berezin M. et al., 2019, ‘Screening and supporting through schools: Educational experiences and needs of adolescents living with HIV in a South African cohort’, *BMC Public Health* 19(1), 272 10.1186/s12889-019-6580-0 30841878 PMC6404343

[CIT0028] UNESCO, 2008, *Good policy and practice in HIV & AIDS and education booklet 2; HIV and supportive learning environments*, 2nd edn, UNESCO, Paris.

[CIT0029] Vaismoradi M., Turunen H. & Bondas T, 2013, ‘Content analysis and thematic analysis: Implications for conducting a qualitative descriptive study’, *Nursing & Health Sciences* 15(3), 398–405. 10.1111/nhs.12048 23480423

[CIT0030] Woolcock M, 2001, ‘The place of social capital in understanding social and economic outcomes’, *Canadian Journal of Policy Research* 2(1), 11–17.

[CIT0031] Zembylas M, 2008, *Practicing an ethic of caring in teaching: Challenges and possibilities. International perspectives on education*, pp. 159–174, Continuum, London.

[CIT0032] Zuilkowski S.S., Fink G., Moucheraud C. & Matafwali B, 2012, ‘Early childhood education, child development and school readiness: Evidence from Zambia’, *South African Journal of Childhood Education* 2(2), 20 10.4102/sajce.v2i2.15

